# Relationship between idiopathic scoliosis and body composition -a case-control study

**DOI:** 10.3389/fphys.2026.1834442

**Published:** 2026-05-15

**Authors:** Andrzej Siwiec, Małgorzata Domagalska-Szopa, Jan Siwiec, Agata Dobrowolska, Andrzej Szopa

**Affiliations:** 1 Child and Family Health Center in Sosnowiec, Sosnowiec, Poland; 2 Department of Developmental Age Physiotherapy, Faculty of Health Sciences in Katowice, Medical University of Silesia in Katowice, Sosnowiec, Poland; 3 Clinical Department of Pediatrics, Faculty of Medical Sciences in Katowice, Medical University of Silesia in Katowice, Sosnowiec, Poland; 4 Department of Physiotherapy, Faculty of Health Sciences in Katowice, Medical University of Silesia in Katowice, Katowice, Poland

**Keywords:** bioelectrical impedance analysis, body mass index, idiopathic scoliosis, OLAF, spinal deformity

## Abstract

**Background:**

This study investigated the association between idiopathic scoliosis (IS) and body composition in Polish children and adolescents compared to healthy peers, using bioelectrical impedance analysis (BIA). IS is a spinal deformity seen in youth, often linked with a thinner build (lower BMI and body weight), but body composition details are poorly documented in Europeans. The study aimed to compare fat, muscle, and other body components between IS patients and controls, and to examine links with scoliosis severity (Cobb angle).

**Methods:**

Cross-sectional design, with 92 IS participants and 94 age- and gender-matched controls. Measurements included BMI, fat and muscle mass, bone mass, metabolic rate, and more via BIA.

**Results:**

The IS group had lower BMI and muscle mass than controls. Despite lower muscle mass, IS subjects had higher body fat percentage, absolute fat mass, and waist-to-height ratio (central fat). Bone mass and metabolic rate showed no significant difference between groups. Greater spinal curvature is linked with lower BMI, muscle mass, and metabolic activity. Implications. IS is associated with a specific body composition: less muscle, higher central fat, and lower BMI.

**Conclusion:**

Consequently, routine body composition assessment in IS management is recommended. Study limitations include its cross-sectional design, which prevents causal inference, a relatively small sample, and unaccounted factors like physical activity. Longitudinal research is needed to clarify these relationships and explore therapeutic body composition modifications.

## 1 Introduction

Idiopathic scoliosis (IS) is the most common spinal deformity in the pediatric population. IS is a complex developmental defect of the spine that leads not only to lateral curvature in the frontal plane, as the name “scoliosis” suggests, but also in the other two anatomical planes. In the sagittal plane, the natural curvatures of the spine are disturbed (shallowing or deepening of physiological kyphosis and lordosis), and in the horizontal plane, there is rotation of the vertebrae around the longitudinal axis (spinal rotation, vertebral torsion) (Weinstein, 2019). The consequence of these changes are secondary trunk deformations, which include shifting, tilting, and torsion of the thorax, resulting in the formation of a rib hump, and/or torsion of the lumbar vertebrae, resulting in the formation of a lumbar roll, as well as lowering and anterior rotation of the iliac crest on the side of the primary curve and hip protrusion. The full picture of scoliosis symptoms is complemented by symptoms extending beyond the spine and its associated segments, such as asymmetry of the head, shoulders, shoulder blades, and waist triangles, and biomechanical changes in the lower limbs (Weinstein, 2019). Previous studies have also shown the occurrence of hormonal disorders (Schlösser et al., 2015), limited respiratory mobility of the chest (Szopa and Domagalska-Szopa, 2017), and reduced cardiopulmonary fitness among people with idiopathic scoliosis (Siwiec et al., 2024). Respiratory impairment, particularly in more severe curves, may further contribute to reduced physical activity and metabolic alterations (Kato et al., 2019).

Although by definition, the cause of IS is unknown, the currently prevailing view is that it is a systemic disease and its pathogenesis results from a complex interaction of genetic or hereditary factors, hormonal disorders, metabolic abnormalities (including systemic osteopenia), and biomechanical and environmental factors (Menger and Sin, 2025).

There is currently an ongoing debate in the literature regarding the relationship between IS and changes in body composition in individuals with it. Previous research has shown that children and adolescents with IS tend to share a distinct anthropometric profile, characterized by lower body weight, reduced BMI, and a higher prevalence of underweight status (Hershkovich et al., 2013; Cheuk et al., 2015; Tarrant et al., 2015; Ciaccia et al., 2017; Zheng et al., 2017; Kim et al., 2020; Watanabe et al., 2020; Miyagi et al., 2021; Scaturro et al., 2022). Studies employing body composition analysis further indicate that individuals with IS may have decreased bone mineral density (Clark et al., 2014; Cheuk et al., 2015; Wang et al., 2016; Matusik et al., 2020; Miyagi et al., 2021) and, in some reports, reduced fat−free mass (Clark et al., 2014; Wang et al., 2016; Matusik et al., 2020; Miyagi et al., 2021). However, most of this work has been conducted in Asian populations (Wang et al., 2016; Zheng et al., 2017; Kim et al., 2020; Miyagi et al., 2021) and has produced inconsistent findings regarding the distribution of specific body components, particularly muscle and adipose tissue (Clark et al., 2014; Wang et al., 2016; Matusik et al., 2020; Miyagi et al., 2021; Scaturro et al., 2022).

Despite growing evidence of body composition alterations in IS, several important gaps persist. First, there is a paucity of research examining detailed body composition—including simultaneous assessment of muscle, fat, and bone mass—in European pediatric populations with IS, where genetic and environmental factors may differ from Asian cohorts (Scaturro et al., 2022). Second, existing studies have not clearly determined whether the lower body weight in IS reflects proportional reduction of all tissues or a selective alteration in specific components (e.g., muscle deficit with preserved or increased adiposity). Third, the relationship between body composition parameters and clinical features of scoliosis (such as curve severity) remains incompletely understood, particularly in mild-to-moderate IS, where early intervention might be most beneficial.

This study addresses these gaps by providing the first comprehensive assessment of body composition using bioelectrical impedance analysis (BIA) in a well-characterized cohort of Polish children and adolescents with mild to moderate IS, compared to age- and sex-matched healthy controls. Unlike previous reports focusing primarily on Asian populations, this study investigates a European cohort, offering insights into population-specific patterns. By analyzing both absolute and relative measures of muscle mass (PMM), fat mass (FM, BF%), bone mass (BM), and body water distribution (TBW), we aim to determine whether the “lean” phenotype in IS conceals a more complex metabolic profile characterized by muscle deficit and simultaneous fat accumulation. Furthermore, by examining correlations between body composition parameters and clinical features (Cobb angle, Risser sign), we seek to identify potential associations that may inform future prognostic and therapeutic strategies.

Taking the above into account, the main aim of this study was to thoroughly assess and compare body composition (including fat, muscle, bone, and lean body mass) in Polish children and adolescents with IS and their healthy peers using bioelectrical impedance analysis (BIA). The specific objectives were: (1) to identify possible differences in body composition between the study and control groups; (2) to identify which body mass components are linked to the presence of IS; (3) to assess the relationship between key body composition parameters and clinical features of scoliosis, including the Cobb angle.

## 2 Materials and methods

This is a cross-sectional study. The reporting of this study conforms to the STROBE guidelines for case-control studies (Vandenbroucke et al., 2007). The study design, protocol, and consent forms were developed in accordance with the Code of Ethics of the World Medical Association (Declaration of Helsinki). This study was approved by the Ethical Committee of the Medical University of Silesia (Resolution No. (BNW/NWN/0052/KB1/93/25) on 26 August 2025). Before each child’s examination, a parent or guardian was informed about the course of the study and the potential risks. Furthermore, the parents or guardians provided written informed consent. Apart from the consent of their parents/legal representatives or guardians, adolescents aged 16 years who acted as minors were required to independently grant their own consent.

Before starting the study, the minimum sample size was calculated using G*power 3.1.9.4. The *a priori* power analysis (f² = 0.15, α = 0.05, power = 0.95) indicated a minimum of 90–95 participants per group. With N = 186, the study was adequately powered; *post hoc* analysis confirmed sufficient power (0.81) even for the smallest significant effects observed (BF% and FM models, f² = 0.06 (Hoenig and Heisey, 2001).

### 2.1 Subjects

Ninety-two consecutive children and adolescents (mean age 13.30 years, range 10–17 years) with mild and moderate scoliosis, Idiopathic Scoliosis (IS), attending local pediatric rehabilitation centers and participating in rehabilitation camps were included in the current study. Patients were diagnosed with idiopathic scoliosis based on the Cobb angle measured on standing posteroanterior radiographs in accordance with internationally accepted criteria (Menger and Sin, 2025). All X-rays were obtained as part of routine clinical monitoring of scoliosis progression by a board-certified radiologist who remained unaware of the project’s aims and the patients’ clinical data. The radiological reports and Cobb angle measurements were subsequently reviewed and confirmed by an experienced orthopedic surgeon before final inclusion of the participant. This approach was chosen to avoid unnecessary additional radiation exposure in this pediatric population.

The inclusion criteria were as follows: (1) children and adolescents (both male and female) aged 10–17; (2) with mild -to moderate scoliosis (Cobb angle on X-ray radiograph within the range of 10–40°); (3) able to follow study instructions. The diagnosis of IS was confirmed radiologically, defined by a Cobb angle >10°. Subjects with a history of connective tissue abnormalities, neuromuscular diseases, intellectual disability, or other congenital spinal deformities were excluded.

Exclusion criteria: 1) Cobb angle > 40°; 2) acute and chronic conditions other than scoliosis; 3) lack of consent from the child’s legal guardians.

The control group included 94 gender- and age-matched healthy controls who were students at a local primary school. Exclusion criteria were: 1) lack of consent from the subject/parents/legal guardians to participate in the study; 2) scoliosis or other diagnosed deformities of the spine and thorax.

Each additional individual who met the inclusion criteria for the study groups was included in the study. Characteristics of the subjects in both groups and comparisons between the groups are included in **Table 1**.

**Table 1:** Demographic and anthropometric data of the study (n = 92) and control (n = 94) groups, including between-group comparisons and characteristics of scoliosis in the study group.

Parameters	Study group	Control group	t; p
Age (years); M ± SD; range	13.30 ± 2.46; 10–17	13.26 ± 2.42; 10–17	0.68;.74
Height (cm); M ± SD; range	156.00 ± 15.55; 131–182	159.05 ± 12.16; 135–185	0.87;.31
Weight (kg); M ± SD; range	45.61 ± 17.00; 32.7–95.0	47.49 ± 15.12; 33.7–95	0.62;.77
Z−BMI; M ± SD; range	0.7 ± 1.3; -1.8–2.0	1.2 ± 0.8; -2.0 –2.0	-6.25; <.001
Girls; N (%)	50 (53.2%)	48 (52.1%)	
Boys; N (%)	44 (46.8%)	44 (47.8%)	
Raimondi test; M ± SD; range	9.92 ± 6.10; 2–30		
Cobb angle (primary); M ± SD; range	20.90 ± 11; 11–40		
Cobb angle (secondary); M ± SD; range	15.83 ± 8.87;3–33		
RT 0; N (%)	8 (8.7%)		
RT 1; N (%)	20 (21.7%)		
RT 2; N (%)	15 (16.3%)		
RT 3; N (%)	14 (15.2%)		
RT 4; N (%)	27 (29.3%)		
RT 5; N (%)	8 (8.7%)		

M, mean; SD, standard deviation; range, minimum–maximum; t, t-test value; p, p-value; RT, Risser test.

### 2.2 Testing procedure

Each participant was examined on one day and the examination lasted approximately 60 minutes. The study was a one-time study and consisted of three parts: 1) medical record analysis and radiographic measurements; 2) anthropometric measurements and BMI percentile calculation (**Table 2**); and 3) Total and Segmental Body Composition Examination.

**Table 2:** Anthropometric indicators.

Indicator	Abbreviation	Definition
OLAF Body Mass Index	O BMI	A Body Mass Index value calculated using Polish growth reference charts specific to the Polish pediatric population
OLAF height percentile	O Ht pctl	A measurement of a child’s height as part of the OLAF database. This is an anthropometric measurement used for growth moni-toring and, in combination with weight, for calculating the OLAF BMI.
OLAF weight percentile	O Wt pctl	A measurement of a child’s weight as part of the OLAF database. This is a fundamental anthropometric measurement used for growth monitoring and, in combination with height, for calculating the OLAF BMI.
Z-score Body Mass Index	Z−BMI	A statistical measure indicating how many standard deviations an individual’s BMI is from the average BMI for their age and gender in a reference population.
Waist-to-Height Ratio	WHtR	A ratio calculated by dividing the waist circumference by the person’s height.

Based on the radiographic measurements, the following indices were calculated: 1) Cobb angle (primary and secondary curvatures); and then, the scoliosis curve with the greatest magnitude of Cobb angle was defined as the major curve; 2) the degree of ossification of the iliac apophysis by X-ray, evaluating for the Risser sign, a 5-point staging scale for assessing bone maturation and 3) the degree of scoliotic vertebral rotation of the major curve according to the Raimondi method.

Bioelectrical impedance analysis (TANITA MC-780 S MA) was chosen for body composition assessment. While DXA is the reference method, BIA was selected based on strong validation, practical feasibility, and ethical considerations. Recent large-scale studies demonstrate excellent correlation between multi-frequency BIA and DXA for muscle and fat mass, with concordance coefficients of 0.94 (Feng et al., 2024; Ha et al., 2024). Moreover, the identical TANITA MC-780 device has been specifically validated in idiopathic scoliosis research” (Miyagi et al., 2021).

Before the body composition examination, each subject’s height was measured using a TANITA height gauge with an accuracy of 0.5 cm. Then, a Total and Segmental Body Composition Examination (respectively TBC; SBC) was performed using a TANITA MC-780 S MA scale (TANITA Corporation, Japan, in Tokyo (1-14–2 Maen-ocho, Itabashi-ku, Tokyo 174–8630 Japan). During the examination, the patient was undressed down to their underwear and stood erect on the scale in a relaxed standing position. All examinations were conducted around noon (between 11:00 a.m. and 1:00 p.m.), after the subjects had eaten a light breakfast at around 9:00 a.m. but before lunch. All measurements were performed by a single trained researcher with extensive experience in bioelectrical impedance analysis and standardized anthropometric assessment, ensuring consistency across all participants.

Based on the obtained data, general body mass indices were calculated: BMI percentile and Z−BMI using the AntroPlus program (in accordance with the recommendations of the World Health Organization (WHO) (Ha et al., 2024; World Health Organization, n.d.) and BMI OLAF (O BMI; O Ht pctl, O Wt pctl), percentile charts developed by the Children’s Memorial Health Institute) (Children’s Memorial Health Institute, n.d.; Kułaga et al., 2011).

The following body composition parameters were included in the further analysis: BMR, BF%; FM, FFM, percentage of TBW%, PMM, BM, and PhA (**Table 3**).

**Table 3:** Body composition parameters measured by bioelectrical impedance analysis (BIA).

Indicator	Abbreviation	Definition	Unit
Basal Metabolic Rate	BMR	The number of calories the body needs at rest to maintain basic physiological functions.	kJ
Body Fat Percentage	BF%	The proportion of a person’s total body weight that is composed of fat tissue.	%
Fat Mass	FM	The absolute weight of fat tissue in the body.	Kg
Fat-Free Mass	FFM	The total weight of all body components except fat, including muscles, bones, organs, and water.	Kg
Total Body Water Percentage	TBW%	The percentage of total amount of water in the body, comprising both intracellular and extracellular water.	%
Predicted Muscle Mass	PMM	An estimate of the total mass of skeletal muscle in the body.	Kg
Bone Mass	BM	The estimated mineral mass (primarily calcium) in the bones.	Kg
Phase Angle	PhA	A measurement from BIA that reflects cellular integrity and health; a higher value generally indicates better cell function.	kHz

### 2.3 Statistical analysis

In the first step of data analysis, basic descriptive statistics were performed, including a test of normality using the Kolmogorov-Smirnov test. All analyses below were performed using SPSS 29.0 statistical software, and the level of statistical significance was set at p < 0.05. Analysis of the variable distributions revealed that they did not conform to a normal distribution, except for BMI and BF%. Analysis of the normal distribution also identified outliers and extreme values. Values that deviated from the normal distribution by three times the interquartile range were replaced by the median.

In the next step of the statistical analysis, comparisons were made between the groups regarding demographic parameters, general body mass indexes, and individual body composition components. Because preliminary comparative analyses revealed a significant interactive effect of age and gender on the body composition of the participants, hierarchical regression analysis was performed in the subsequent analysis. Group membership was coded as follows: scoliosis group = 1, control group = 0. Therefore, a negative regression coefficient (B or β) indicates lower values in the scoliosis group compared to controls, while a positive coefficient indicates higher values in the scoliosis group. All regression models were adjusted for age and gender.

Finally, to determine whether clinical features of scoliosis were associated with individual body mass indices, a series of pairwise Pearson r correlation analyses was performed. Although some variables exhibited non-normal distributions, Pearson correlation was used due to the large sample size (n > 90 per group), which ensures the robustness of parametric methods under the central limit theorem (Children’s Memorial Health Institute, n.d.; Ghasemi and Zahediasl, 2012). Pearson correlation is appropriate for assessing linear associations between continuous clinical variables and body composition parameters, and its use is consistent with the regression analyses performed.

## 3 Results

Although the compared groups (cases and controls) are homogeneous in terms of gender and age distribution (**Table 1**), preliminary analyses revealed a significant interactive effect of age and gender on the body composition of the subjects (**Tables 4**, **5**). In the next step of the analyses, we decided to check to what extent the group of cases = 1 vs. controls = 0) allows for explaining the variability in individual body weight parameters.

**Table 4:** Summary of weight and height measurements in the study (n = 92) and control (n = 94) groups.

Parameters	Study group	Control group	t; p
	M ± SD		
Z−BMI	0.7 ± 1.30	1.80 ± 1.99	-6.25; <.001
O BMI	19.28 ± 3.27	21.41 ± 7.92	-2.39;.018
O Ht pctl	50.53 ± 29.57	50.47 ± 31.63	0.01;.989
O Wt pctl	47.28 ± 31.59	64.51 ± 32.78	-3.65; <.001
WHtR	0.50 ± 0.07	0.44 ± 0.06	5.47; <.001

M, mean; SD, standard deviation; t, t-test value; p, p-value.

**Table 5:** Summary of body composition data (Tanita test) in the study (n = 92) and control (n = 94) groups.

Parameters	Study group	Control group	t; p
	M ± SD		
BMR	5633.89 ± 1312.9	5894.71 ± 1109.8	-1,46;.146
BF%	26.92 ± 8.08	22.96 ± 5.78	3.84; <.001
FM	13.00 ± 8.03	12.12 ± 6.51	0.82;.414
FFM	33.23 ± 11.61	38.14 ± 9.24	-3.19;.002
TBW%	24.61 ± 8.70	27.90 ± 5.99	-2.87;.005
PMM	31.97 ± 11.03	35.99 ± 8.99	-2.73;.007
BM	1.75 ± 0.55	1.97 ± 0.44	-3.07;.002
PhA	5.21 ± 0.51	5.26 ± 0.47	-0.76;.447

M, mean; SD, standard deviation; t, t-test value; p, p-value.

The model also controlled for gender (coded as female = 0, male = 1) and age. Detailed results of the stepwise regression model are presented in **Tables 6**, **7**. Two linear regression analyses were conducted to determine whether age, gender, and group membership (with vs without scoliosis) were predictors of overall body mass, as measured by the O- BMI and Z-BMI indices (**Table 6**), and whether they were predictors of body mass composition, as measured by individual parameters (**Table 7**).

**Table 6:** Coefficients of multiple linear regression for the associations between group, age, gender, and body composition parameters.

Dependent Variables	Predictors	B	SE	β	t	p	95% CI for B		R^2^
							LL	UL	
O BMI	(Constant)	15.89	2.16		7.35	<.001	11.62	20.15	0.14**
	Age (years)	0.35	0.11	0.27	3.31	.001	0.14	0.56	
	Gender (male=1)	- 2.05	0.54	-0.26	-3.78	<.001	-3.12	-0.98	
	Group (scoliosis=1)	-2.23	0.51	-0.39	-4.37	<.001	-3.23	-1.23	
Z−BMI	(Constant)	2.31	0.79		2.91	.004	0.74	3.87	0.27**
	Age (years)	-0.13	0.04	-0.24	-3.24	.001	-0.20	-0.05	
	Gender (male=1)	0.81	0.20	-0.26	-4.06	<.001	-1.20	-0.42	
	Group (scoliosis=1)	-2.70	0.63	-0.33	-4.29	.001	-3.94	-1.46	
WHtR	(Constant)	0.56	0.04		14.97	<.001	0.49	0.63	0.27**
	Age (years)	-0.005	0.002	-0.20	-2.70	.008	-0.009	-0.001	
	Gender (male=1)	-0.05	0.01	-0.32	-4.90	<.001	-0.07	-0.03	
	Group (scoliosis=1)	0.03	0.01	0.27	3.00	.003	0.01	0.05	

B, unstandardized coefficient; SE, standard error; β, standardized beta; t, t-value; p, significance level; LL, lower confidence limit; UL, upper confidence limit for B; R², adjusted R-squared; gender (male); group (scoliosis). **p <.001.

**Table 7:** Linear regression coefficients for body composition parameters.

Dependent variable	Predictors	B	SE	β	t	p	95% CI for B		R^2^
							LL	UL	
BMR	(Fixed)	4972.63	569.93		8.72	<.001	3848.10	6097.16	.42**
	age	218.33	28.06	0.51	7.78	<.001	162.97	273.69	
	gender	-1254.02	143.30	-0.50	-8.75	<.001	-1536.76	-971.28	
	group	-170.98	162.60	-0.07	-1.05	.294	491.80	149.83	
BF%	(fixed)	18.19	4.34		4.20	<.001	9.64	26.75	.06*
	age	0.11	0.21	0.04	0.49	.621	-0.32	0.53	
	gender	-0.49	1.09	-0.03	-0.45	.656	-2.64	1.66	
	group	4.20	1.24	0.39	3.39	<.001	1.76	6.64	
FM	(fixed)	3.21	4.35		0.74	.461	-5.36	11.78	.06*
	age	0.72	0.21	0.29	3.39	<.001	0.30	1.15	
	gender	-2.01	1.09	-0.13	-1.84	.067	-4.17	0.15	
	group	2.71	1.24	0.29	2.19	.030	0.27	5.16	
FFM	(fixed)	11.21	4.58		2.45	.015	2.17	20.25	.52**
	age	2.76	0.23	0.74	12.22	<.001	2.31	3.20	
	gender	-6.94	1.15	-0.31	-6.03	<.001	-9.22	-4.67	
	group	-2.19	1.31	-0.10	-1.67	.102	-4.77	0.37	
TBW%	(fixed)	0.45	0.03		13.15	<.001	0.38	0.51	.006
	age	<.001	0.002	0.04	0.44	.663	-0.003	0.004	
	gender	0.01	0.01	0.10	1.38	.170	-0.005	0.03	
	group	0.02	0.01	0.15	1.66	.099	-0.003	0.04	
PMM	(fixed)	9.37	4.34		2.16	.032	0.81	17.93	.53**
	age	2.67	0.21	0.75	12.49	<.001	2.25	3.09	
	gender	6.82	1.09	-0.32	-6.25	<.001	-8.97	-4.67	
	group	-2.83	1.24	-0.34	-2.29	.023	-5.27	-0.39	
BM	(fixed)	0.86	0.21		4.04	<.001	0.44	1.29	.53**
	age	0.13	0.01	0.72	12.08	<.001	0.11	0.15	
	gender	-0.39	0.05	-0.37	-7.27	<.001	-0.50	-0.29	
	group	-0.09	0.06	-0.09	-1.48	.142	-0.21	0.03	
PhA	(fixed)	4.62	0.29		15.86	<.001	4.05	5.20	.05*
	age	0.04	0.01	0.26	3.07	.002	0.02	0.07	
	gender	-0.10	0.07	-0.10	-1.37	.172	-0.25	0.04	
	group	0.17	0.08	0.18	2.04	.042	0.01	0.33	

B, unstandardized coefficient; SE, standard error; β, standardized beta; t, t-value; p, statistical significance; LL, lower limit; UL, upper limit for B (95% CI); R², adjusted R-squared; gender (male); group (scoliosis).*p <.05, **p <.001.

In the regression model for general body mass indices, it was observed that the group factor explains the variability in general body mass expressed by the O -BMI and Z−BMI indices in a negative and moderate way (β = -0.39 β = -0.33, respectively). The proposed regression model explaining the O- BMI and Z−BMI variables fits the data well, O -BMI.

F(3, 182) = 8.77, p <.001 and Z−BMI F(3, 182) = 8.50, p <.001, and allows for explaining 14% of the variability in the O- BMI parameter and 27% of the variability in the Z−BMI parameter.

Since the equations of the proposed regression model are as follows: O-BMI = 15.89 + 0.35 × (age in years) + 2.05 × (gender – male) − 2.23 × (scoliosis group) and Z-BMI = 2.31 + 0.13 × (age in years) + 0.81 × (gender – male) − 2.70 × (scoliosis group), this means that children with scoliosis are characterized by an average lower O-BMI by 2.23 and a lower Z−BMI by 2.70, regardless of the age and gender of the subjects. In the regression analysis for both body mass index values, age and gender had a significant positive effect (β = 0.27 for O -BMI and β = 0.24 for Z-BMI, both p <.05), β = 0.26 for both, p <.05). This means that both O- BMI and Z−BMI increased with age and were significantly higher in boys compared to girls. The regression model for WHtR (waist-to-height ratio) was also statistically significant, explaining 27% of the variability in this parameter. However, analysis of the results obtained here showed that the group of participants with scoliosis had a significantly higher WHtR (by 0.03 units) compared to the control group.

Analyzing the results for body mass components in the regression model, we observed that the group factor (scoliosis/non-scoliosis) significantly explained the variability in several key parameters. Belonging to the “scoliosis” group explained the variability in PMM negatively and moderately (β = -0.34). The proposed regression model fitted the data very well, F(3, 182) = 68.76, p <.001, and explained as much as 53% of the variance of the dependent variable, which makes it the strongest model in the conducted analysis. According to the equation of this model (PMM = 9.37 + 2.67 × age + 6.82 × gender − 2.83 × group), this means that the subjects with scoliosis were characterized by an average lower level of PMM by 2.83 units, regardless of their age and gender. It is worth emphasizing here that although the presence of scoliosis was a significant predictor, the strongest factor explaining the level of PMM was the age of the subjects (β = 0.75), which means that the younger the subject, the lower their level of PMM (**Table 7**). Similar, although opposite, trends were observed for both parameters characterizing the content of adipose tissue, i.e. BF% (body fat percentage) and FM (absolute fat mass). The group factor positively and weakly explained the variability of fat mass indices (BF%: β = 0.39; FM: β=0.29). The regression models for adipose tissue parameters were statistically significant, each explaining 6% of the variance (R² = 0.06, p <.05).

The obtained results indicate that among participants with scoliosis, after controlling for age and gender, their body fat levels – both in absolute values (FM) and percentage indices (BF%) – were statistically significantly higher than in their healthy peers, regardless of age and gender (**Table 7**). Furthermore, a significantly higher PhA was found in the group of participants with scoliosis (B = 0.17, p = .042) (**Table 7**).

To check whether the clinical features of scoliosis are related to both the overall body mass index and its individual components, a series of Pearson r correlation analyses was performed. Detailed results of the analysis are presented in **Table 8**. The analysis of the obtained results revealed statistically significant negative correlations between the degree of curvature (Cobbe angle) and general body mass indices, such as O -BMI (r = -0.228, p = .029) and O Wt pctl (r = -0.215, p = .040). Although these correlations are weak, they suggest a general tendency to indicate that scoliosis with a higher degree of severity (expressed by a larger Cobb angle) is accompanied by lower general body mass indices. Further negative correlations (moderate and weak) were found between the primary curvature angle (Cobbe angle) and the BMR (r = -0.304, p = .003) as well as with lean body mass (FFM: r = -0.223, p = .033) and PMM (r = -0.238, p = .022), which may suggest that a greater spinal curvature is accompanied by a reduction in metabolically active body tissues. The analysis also confirmed obvious relationships between the Risser grade (reflecting the skeletal maturity of the subjects) and the overall O -BMI (weak positive correlations; r = 0.247, p = .018), as well as individual components of the body composition of the subjects with scoliosis. These were strong positive correlations with muscle and metabolic parameters such as: PMM (r = 0.427, p <.001), lean body mass (FFM; r = 0.431, p <.001), hydration level (TBW%; r = 0.420, p <.001) and FM (r = 0.307, p = .003) and weaker correlations with the level of BMR (r = 0.219, p = .036) and BF% (r = 0.240, p = .021). The positive pattern of the above correlations only confirms that greater skeletal maturity correlates with better body composition parameters, i.e. higher muscle mass, bone mass, greater lean body mass, but also higher fat mass, which undoubtedly reflects the natural process of growing up and the associated changes in body composition.

**Table 8:** Relationships between individual body mass indices and quantitative characteristics of scoliosis.

Parameters	Pearson’s	Cobb angle	King-Moe	Risser	Raimondi
O BMI	*r*	-.228*	.06	.247*	-.01
	p	.029	.565	.018	.944
O Ht pctl	*r*	-.12	.15	-.10	.02
	p	.263	.141	.365	.827
O Wt pctl	*r*	-.215*	.15	-.14	-.03
	p	.040	.167	.193	.780
Z−BMI	*r*	-.17	.01	-.06	-.04
	p	.111	.897	.587	.715
WHR	*r*	-.07	.03	-.261*	-.08
	p	.489	.799	.012	.455
WHtR	*r*	-.12	.13	-.19	-.12
	p	.243	.231	.077	.254
BMR	*r*	-.304**	.05	.219*	-.08
	p	.003	.663	.036	.446
BF%	*r*	-.04	.03	.240*	.10
	p	.680	.790	.021	.332
FM	*r*	-.11	.02	.307**	.06
	p	.288	.851	.003	.555
FFM	*r*	-.223*	.01	.431**	-.04
	p	.033	.914	<.001	.685
TBW%	*r*	-.238*	.01	.420**	-.03
	p	.022	.918	<.001	.769
PMM	*r*	-.238*	.03	.427**	-.04
	p	.022	.747	<.001	.727
PhA	*r*	-.271**	.04	.08	-.292**
	p	.009	.695	.445	.005

O-BMI, observed body mass index (OLAF); O Ht pctl, observed height percentile (OLAF); O Wt pctl, observed weight percentile (OLAF); Z-BMI, BMI z-score; WHR, waist-to-hip ratio; WHtR, waist-to-height ratio; BMR, basal metabolic rate; BF%, body fat percentage; FM, fat mass; FFM, fat-free mass; TBW%, total body water percentage; PMM, predicted muscle mass; PhA, phase angle; Pearson’s, Pearson correlation coefficient; Cobb angle, degree of spinal curvature; King-Moe, classification of scoliosis curve type; Risser, Risser sign (skeletal maturity); Raimondi, scoliosis classification index; p, p-value; r, Pearson correlation coefficient.

## 4 Discussion

The aim of this study was, among other things, to compare overall body mass indexes in Polish children and adolescents with IS with their healthy peers. Our results fully confirm the presence of a characteristic “lean” anthropometric phenotype in patients with IS, as described in the literature (Zheng et al., 2017; Miyagi et al., 2021; Scaturro et al., 2022). The significantly lower O-BMI and Z−BMI values in the study group compared to the control group are consistent with one of the most consistent findings from many studies demonstrating lower body weight and lower indices based on body weight and height in this population (Zheng et al., 2017; Kim et al., 2020; Scaturro et al., 2022). The results of our regression analysis clearly confirmed that membership in the “scoliosis” group was a strong predictor—independent of age and gender—of lower levels of both O-BMI and Z−BMI. However, confirming the general phenotypic pattern of individuals with IS was only a starting point for a more in-depth comparative analysis of their body composition, with particular emphasis on muscle mass, lean body mass, bone mass, and fat mass. Although the lower overall body weight in individuals with IS has been well documented in numerous studies [7−15], the results regarding individual body components, especially muscle mass and lean mass, as well as bone mass and adipose tissue, are fewer and not entirely consistent [10, 16−18]. While the literature reports reduced skeletal muscle mass (Zheng et al., 2017; Miyagi et al., 2021) and lower lean mass indices, which largely correspond to muscle mass (Wang et al., 2016), the results of this study reveal a more complex picture of body composition disturbances in children and adolescents with IS.

The regression analysis conducted in this study revealed a distinctive body composition in Polish children and adolescents with IS, consisting of three key, interrelated elements.

First, one of the strongest factors differentiating study participants—independent of age and gender—was PMM. Patients with IS had lower PMM levels by as much as 2.83 units compared to their healthy peers. Although this observation is consistent with previous reports by Miyagi et al. (2021) and Wang et al. (2016) and Clark (Clark et al., 2014) who also demonstrated lower muscle mass and lean mass in IS patients, our results also provided evidence for the presence of disorders related to fat metabolism. Despite lower muscle mass, patients with scoliosis had higher percentage body fat (%BF) and absolute FM. The regression model showed that assignment to the study group was associated with an increase in this parameter by 0.10 units. In contrast to some previous studies (Zheng et al., 2017; Kim et al., 2020) which described generalized thinness in children and adolescents with IS, in our cohort, the IS group was characterized by higher BF% and FM. This seemingly paradoxical body composition profile: “lower BMI, reduced muscle mass, and simultaneously increased body fat” is further supported by the results of a regression analysis of WHtR (Waist-to-Height Ratio). WHtR reflects the presence of central (abdominal) obesity and is considered a better indicator of metabolic risk than BMI. Based on the obtained results, the model for WHtR was statistically significant, explaining 27% of the variability in this parameter, indicating that the scoliosis group had a higher WHtR (by 0.03 units) compared to the control group. This suggests that body composition abnormalities in IS are not limited to underweight and reduced muscle mass, but encompass an imbalance between muscle and fat tissue. This means that despite lower total body mass (lower BMI), its distribution in IS patients is less favorable, characterized by a higher proportion of fat tissue relative to muscle mass. This confirms the observations of Clark et al. (2014) that overall body mass, not just its individual components, may play a role in the pathogenesis of IS. A higher, unfavorable WHtR (Waist-to-Height Ratio) in children and adolescents with scoliosis indicates increased central fat accumulation, further underscoring the complexity of body composition alterations in idiopathic scoliosis.

This is confirmed by the altered cellular indicator in the form of PhA, which was significantly higher (by 0.17 units) in the scoliosis group, differing from the values observed in the control group. In the context of simultaneous decreased muscle mass and increased adipose tissue, higher PhA may reflect altered tissue proportions. However, its final interpretation in the context of the specific metabolic profile of IS requires further, in-depth research.

Recognizing the body composition pattern characteristic of IS: “lower BMI, decreased muscle mass, and simultaneously increased adipose tissue” constitutes a - contribution to understanding the nature of body composition abnormalities in IS and confirms previous reports that focusing primarily on overall leanness (Zheng et al., 2017; Kim et al., 2020).

Importantly, the observed phenotype of ‘low BMI, low muscle, but high fat’ reflects more than a simple mathematical consequence of weight loss. The IS group demonstrated significantly higher absolute fat mass (FM) and central adiposity (WHtR) alongside reduced absolute muscle mass (PMM).— This suggests a change involving both muscle wasting and fat accumulation, rather than merely preserved fat mass in the setting of reduced overall body weight. This finding may partially explain the seemingly contradictory reports in the literature, as outlined in the introductory section of this paper.

This body composition pattern, characterized by reduced muscle mass, concomitant increased fat content, and a higher PhA, may be associated with idiopathic scoliosis. It is also worth noting that not all analyzed body composition parameters showed s differences between groups. BM did not differ statistically between groups, nor did body water content (TBW%). Interestingly, the model for total lean mass (i.e., muscle mass, bone mass, and water) was similar in both groups, whereas a statistically significant difference emerged only when the muscle component (PMM) was analyzed alone. All of the above suggest that bone mass and body hydration were not key factors in differentiating between groups. Similarly, despite differences in body composition in terms of muscle and fat mass, the study groups did not differ in terms of resting energy requirements (BRM). To summarize this section of the study, the presented results indicate that, in addition to lower overall body mass indexes (O- BMI, Z−BMI), Polish children and adolescents with IS have a poorer body composition compared to their healthy peers: less active muscle mass and more unfavorable adipose tissue, which is associated with increased long-term health risk. Despite abnormalities in soft tissue composition, the group of participants with IS has normal BM and a normal BMR. The final goal of this study was to assess the relationship between key body composition parameters and clinical features of scoliosis, including the Cobb angle. The results obtained in this study partially confirm and partially complement the current state of knowledge on this topic, while also demonstrating the specificity of the Polish pediatric population. Based on the correlation analysis, statistically significant negative relationships were observed between the Cobb angle and key general body mass indices, as measured by BMI. Despite the weak strength of these correlations (O-BMI: r = -0.228; O Wt pctl: r = -0.215), they indicate a general tendency for more severe scoliosis to be accompanied by lower overall body mass indices. This observation is confirmed by numerous scientific reports [Miyagi et al., Kim et al., Clark et al., 9, 10, 18], and the results of our study are consistent with current research, which confirms the association between low body mass indices and the occurrence and progression of scoliosis. However, although – as indicated by the latest review by Scaturro et al. (2022) – 7 of 10 analyzed studies confirm the association between low BMI and scoliosis, there are also studies demonstrating an association between high BMI and the occurrence of severe spinal curvatures (Margalit et al., Matusik et al.). Therefore, the association between BMI-based indices and scoliosis remains controversial in the literature. In this context, the results of this study, which take into account not only general body mass indices but also specific components of body composition, reveal a more complex relationship between scoliosis and nutritional status. They suggest that it is not so much general body mass but rather the specific distribution of muscle and fat tissue that characterizes individuals with idiopathic scoliosis.

The pattern of significant correlations between clinical features of scoliosis and individual components of body composition obtained in the statistical analysis allowed us to observe several relationships. First, correlation analysis between body composition parameters and the Cobb angle indicated a relationship between the progression of the curve and body composition. The negative correlations were observed between the Cobb angle and meterBMR (r = -0.304), lean body mass (FFM: r = -0.223), and PMM (r = -0.238). These results suggest that scoliosis progression (higher Cobb angle values) appears to be associated with decreased muscle mass and a worsening metabolic parameters in IS participants. These observations are supported by a recent study by Miyagi et al. (2021), which showed that girls with severe scoliosis (Cobb angle >40°) had lower muscle mass index values than those with moderate scoliosis. Similarly, Wang et al. (2016) reported significantly lower lean mass indices in boys with IS compared to their healthy peers. The correlation between a larger Cobb angle and lower PMM and FFM observed in our study further strengthens these findings, suggesting that muscle tissue deficits may be associated with scoliosis progression. Although this specific body composition profile can be both a cause and a consequence of scoliosis progression, it is an important element in understanding the systemic nature of idiopathic scoliosis. On the one hand, reduced muscle mass may contribute to scoliosis progression by weakening the muscular stabilization of the spine. On the other hand, scoliosis progression, through biomechanical changes and potential limitations in physical activity, may lead to further loss of muscle mass, creating a feedback loop. This concept of a vicious cycle is supported by recent biomechanical models of AIS pathogenesis, which emphasize the interaction between passive (skeletal) and active (muscular) subsystems (Li et al., 2025). The positive pattern of correlations between the Risser score and body composition parameters such as muscle, bone, fat, and lean body mass supports the expected positive influence of the natural process of growing up on favorable changes in body composition. The results of this study support the hypothesis that idiopathic scoliosis is accompanied by a distinctive body composition phenotype. However, this study broadens current knowledge on body composition in idiopathic scoliosis in several key ways. While prior research has documented lower body weight and BMI in IS, our findings reveal a more detailed pattern of body composition that has not been previously described in a European pediatric cohort. Specifically, we show that Polish children with IS not only have reduced muscle mass (PMM) but also higher absolute fat mass (FM) and central adiposity (WHtR) compared with healthy peers. To our knowledge, this is the first study to identify such pattern in adolescents with idiopathic scoliosis, emphasizing the importance of re-evaluating nutritional assessment and intervention strategies in this population. These findings suggest that body composition assessment may be useful in the routine monitoring of patients with IS. However, our conclusions apply primarily to children and adolescents with mild-to-moderate IS (Cobb angle 10–40°) from Poland. Wider claims about a metabolic profile of IS are not supported by the present data.

## 5 Conclusions

Further research is needed to explore the potential relationship between body composition modifications and the clinical course of scoliosis Whether modifying body composition could influence scoliosis progression remains an unproven hypothesis requiring longitudinal studies.

## 6 Study limitation

This study, despite providing valuable information on the relationship between body composition and IS, has several limitations that should be considered when interpreting the results. First, the study was conducted on a relatively small study group, purposefully selected from local rehabilitation centers in Poland, which may limit the representativeness of the results and the generalizability of the conclusions to the entire population of children and adolescents with IS. Second, the cross-sectional nature of the study, which prevents us from establishing a cause-and-effect relationship between body composition abnormalities and scoliosis. Therefore, it is impossible to determine whether the observed abnormalities in body composition are the cause, result, or concurrent manifestation of the common risk factor in IS. Third, although we implemented standardized measurement conditions (consistent time of day, fixed interval since the last meal, no strenuous exercise), we did not use direct hydration markers, such as urine specific gravity or osmolality, to confirm comparable hydration status between groups”. Four, it should be noted that bone mass (BM) as measured by BIA represents an estimate rather than a direct measurement of bone mineral content. The absence of significant differences in BM between groups should therefore be interpreted with caution, as it may reflect the methodological insensitivity of BIA rather than true biological equivalence. Future studies using DXA are needed to definitively evaluate bone status in this population.

The final limitation is the lack of consideration of other potentially important factors in the analysis that could modify the relationship between body composition and scoliosis, such as the level and type of physical activity or the participants’ specific dietary habits.

## Data Availability

The raw data supporting the conclusions of this article will be made available by the authors, without undue reservation.
